# The LDL Receptor-Related Protein 1 (LRP1) Regulates the PDGF Signaling Pathway by Binding the Protein Phosphatase SHP-2 and Modulating SHP-2- Mediated PDGF Signaling Events

**DOI:** 10.1371/journal.pone.0070432

**Published:** 2013-07-26

**Authors:** Julie Craig, Irina Mikhailenko, Nathaniel Noyes, Mary Migliorini, Dudley K. Strickland

**Affiliations:** 1 Center for Vascular and Inflammatory Diseases and; 2 Department of Physiology, University of Maryland School of Medicine, Baltimore, Maryland, United States of America; 3 Department of Surgery, University of Maryland School of Medicine, Baltimore, Maryland, United States of America; Hungarian Academy of Sciences, Hungary

## Abstract

**Background:**

The PDGF signaling pathway plays a major role in several biological systems, including vascular remodeling that occurs following percutaneous transluminal coronary angioplasty. Recent studies have shown that the LDL receptor-related protein 1 (LRP1) is a physiological regulator of the PDGF signaling pathway. The underlying mechanistic details of how this regulation occurs have yet to be resolved. Activation of the PDGF receptor β (PDGFRβ) leads to tyrosine phosphorylation of the LRP1 cytoplasmic domain within endosomes and generates an LRP1 molecule with increased affinity for adaptor proteins such as SHP-2 that are involved in signaling pathways. SHP-2 is a protein tyrosine phosphatase that positively regulates the PDGFRβ pathway, and is required for PDGF-mediated chemotaxis. We investigated the possibility that LRP1 may regulate the PDGFRβ signaling pathway by binding SHP-2 and competing with the PDGFRβ for this molecule.

**Methodology/Principal Findings:**

To quantify the interaction between SHP-2 and phosphorylated forms of the LRP1 intracellular domain, we utilized an ELISA with purified recombinant proteins. These studies revealed high affinity binding of SHP-2 to phosphorylated forms of both LRP1 intracellular domain and the PDGFRβ kinase domain. By employing the well characterized dynamin inhibitor, dynasore, we established that PDGF-induced SHP-2 phosphorylation primarily occurs within endosomal compartments, the same compartments in which LRP1 is tyrosine phosphorylated by activated PDGFRβ. Immunofluorescence studies revealed colocalization of LRP1 and phospho-SHP-2 following PDGF stimulation of fibroblasts. To define the contribution of LRP1 to SHP-2-mediated PDGF chemotaxis, we employed fibroblasts expressing LRP1 and deficient in LRP1 and a specific SHP-2 inhibitor, NSC-87877. Our results reveal that LRP1 modulates SHP-2-mediated PDGF-mediated chemotaxis.

**Conclusions/Significance:**

Our data demonstrate that phosphorylated forms of LRP1 and PDGFRβ compete for SHP-2 binding, and that expression of LRP1 attenuates SHP-2-mediated PDGF signaling events.

## Introduction

Despite significant advances in the treatment of severe coronary artery blockage, restenosis continues to represent a serious clinical problem by impeding long-term success of vascular interventions [1]. Restenosis is the process by which an artery treated for occlusion subsequently renarrows due to neointimal formation. This process involves significant vascular remodeling that results from excessive deposition of matrix proteins and from migration and proliferation of vascular SMC (SMC) [2] due to activation of the PDGF signaling pathway [3]. PDGF is a potent mitogen for fibroblasts and SMC, and genetic deletion of either *Pdgfb* or *Pdgfrb* in mice leads to an almost complete lack of pericytes in certain vascular beds [4], [5] confirming a critical role for PDGF-B and the PDGFRβ in vascular smooth muscle cell and pericyte biology. This has been substantiated in experiments which have demonstrated a prominent role for this signaling pathway in vascular remodeling. Thus, balloon catheterization of rat carotid arteries results in increased expression of activated PDGF receptors in the vessel wall [6], [7], and the intimal thickening that follows this treatment is inhibited by administration of neutralizing PDGF antibodies [8]. Further, infusion of PDGF-BB into rats after carotid injury [9], or the expression of recombinant PDGF-BB in porcine arteries [10], caused a significant increase in thickening of the vessel wall due to smooth muscle cell proliferation and matrix deposition by these cells [3].

Both *in vivo* and *in vitro* studies reveal that the LDL receptor-related protein 1 (LRP1) is a physiological modulator of the PDGF signaling pathway. LRP1 is abundantly expressed in vascular SMC, and is a large endocytic and signaling receptor that mediates the endocytosis and subsequent degradation of several ligands including apoE-rich lipoproteins, proteases, and protease-inhibitor complexes [11], [12]. A tissue-specific deletion of the *Lrp1* gene in vascular SMC (smLRP1−/−) on a background of LDL receptor deficiency, causes smooth muscle cell proliferation, aneurysm formation, and a significant increase in susceptibility to cholesterol-induced atherosclerosis [13]. These effects could be inhibited by treatment of the mice with Gleevec, a known inhibitor of tyrosine kinases, including the PDGFRβ. Interestingly, smLRP1(−/−) mice expressed large amounts of activated PDGFRβ in the vessel wall when compared to control LRP1 expressing mice [13]. Overall, the experiments indicate that LRP1 plays an important role in protecting the integrity of the vascular wall and preventing atherosclerosis by suppressing PDGFR activation.

The mechanisms by which LRP1 modulates the PDGF signaling pathway are not well understood. Tight regulation of the PDGFRβ is critical, as excessive activation induces tumor formation [14] and in the vasculature contributes to the development of occlusive vascular disease, such as atherosclerosis and restenosis [2], [3], [6]–[9]. LRP1 co-immunoprecipitates with phosphorylated forms of the PDGFRβ [15] which mediates the tyrosine phosphorylation of the LRP1 intracellular domain (ICD) at tyrosine 4507 within its proximal NPxY motif [16]. This event occurs within endosomal compartments [17], and generates LRP1 molecules with increased affinity for adaptor proteins containing phospho-tyrosine binding (PTB) domains or Src homology 2 (SH2) domains involved in signaling pathways such as Shc [18] and SHP-2 [19], [20].

SHP-2 is a ubiquitously expressed, cytoplasmic protein tyrosine phosphatase (PTP) that contains two SH2 domains [21]. The activity of SHP-2 contrasts the actions of most protein tyrosine phosphatases which negatively regulate signaling pathways by opposing the effects of protein tyrosine kinases [22]. Upon PDGF-stimulation of cells, SHP-2 is recruited to tyrosine residues 763 and 1009 within the PDGFRβ cytoplasmic tail and promotes downstream signaling [22], [23]. Mutation of these two tyrosine residues to phenylalanine generates a receptor that fails to bind SHP-2 and has a significantly reduced chemotaxis response induced by PDGF-BB, revealing an important role for SHP-2 in PDGF-mediated chemotactic signaling [23].

Since phosphorylated forms of LRP1 and the PDGFRβ are both capable of binding SHP-2, we hypothesized that these two receptors may compete for SHP-2 binding, and if so, we reasoned that this may represent one mechanism by which LRP1 suppresses PDGFRβ-mediated signaling. Our results reveal that phosphorylated forms of the LRP1 cytoplasmic domain and the PDGFRβ kinase domain (KD) bind with very high affinity to SHP-2, and compete with one another for this interaction. Further, we observed that SHP-2 co-immunoprecipitates and colocalizes with LRP1 following PDGF stimulation of cells, and that LRP1 attenuates SHP-2 mediated cell migration. Taken together, the data reveal a critical role for LRP1 in modulating SHP-2-mediated PDGF signaling events.

## Materials and Methods

### Cells, Antibodies and Reagents

Newborn rat kidney (NRK) fibroblasts, human WI-38 fibroblasts and murine aortic smooth muscle (MOVAS) cells were purchased from ATCC. LRP1^−/−^ (PEA-13) and B41 clones (LRP1^−/−^ deficient cells transfected with human LRP1) have been described [24]. SHP-2 antibodies were purchased from Santa Cruz Biotechnology (sc-7384, sc-280). SHP-2 phosphotyrosine 542 and SHP-2 phosphotyrosine 580 antibodies were purchased from Cell signaling Technology (3751, 3703). Anti phosphotyrosine antibodies were purchased from BD Transduction laboratories (610000). Monoclonal antibody 8G1 against LRP1 has been previously described [25]. NSC-87877, a potent, small molecule SHP2 inhibitor [26] was obtained from Millipore. The LRP1 intracellular domain (LRP1-ICD) was prepared as a fusion protein with glutathione *S*-transferase as described [27]. GST:LRP1-ICD was phosphorylated using c-src kinase (Upstate, 14–117) in the presence of ATP. Phosphorylation was confirmed by immunoblot analysis using anti-phosphotyrosine antibodies. The PDGFRβ kinase domain (KD) was purchased from Millipore (14–463), and was phosphorylated by incubating the protein with 1 mM ATP and 1 mM MgCl_2_. Soluble PDGFRβ ectodomain (sPDGFr) was purchased from Sino Biological, Inc.

### Pull Down Experiments

For pull-down experiments, NRK fibroblasts were grown to 70% confluence on 60 mm dishes. Cells were lysed in lysis buffer (1%NP40, 1∶100 phosphatase inhibitor cocktail, set II (Calbiochem, 524625), 2 mM activated sodium orthovanadate, 1 mM PMSF, 0.3 µM okadaic acid, protease inhibitor cocktail tablets (Roche Diagnostics), in TBS) and cell lysates were incubated with phosphorylated or unphosphorylated GST:LRP1-ICD immobilized on Glutathione-Sepharose. Following incubation and washing, proteins were eluted and separated by SDS-PAGE and analyzed by immunoblot analysis using the indicated antibodies.

### Immunoprecipitation and Immunoblot Analysis

Prior to lysis, cells were incubated for 30 minutes on ice with the cell-permeable crosslinking agent, DSP (dithiobis[succinimidylpropionate]) (Pierce Biotechnology, Inc.). Following incubation, cells were washed and then collected in lysis buffer (1% NP40, 1∶100 phosphatase inhibitor cocktail, set II (Calbiochem, 524625), 2 mM activated sodium orthovanadate, 1 mM PMSF, 0.3 µM okadaic acid, protease inhibitor cocktail tablets (Roche Diagnostics), in TBS). Lysates were incubated with Dynabead Protein G (Invitrogen, 1007D) overnight at 4°C. Immunoprecipitates were washed, separated by SDS-PAGE on 4–12% tris-glycine precast gels (Invitrogen), and transferred to nitrocellulose membranes for immunoblot analysis. Whole cell lysates were separated by SDS-PAGE on 4–12% tris-glycine precast gels (Invitrogen) and then electrophoretically transferred to nitrocellulose membranes. Immunoblots were first incubated for 1 hour at room temperature in buffer containing 50 mM Tris, 150 mM NaCl, 0.1% Tween 20, and 5% nonfat dry milk. The membranes were then incubated overnight with specific antibodies and washed in buffer containing 50 mM Tris, 150 mM NaCl, and 0.1% Tween 20. Antibody binding to the immunoblots was detected by incubation with an appropriate IRDye® (LI-COR Biosciences) -conjugated secondary antibody. Immunoreactive bands were detected using LI-COR Odyssey Infrared Imaging System.

### ELISA

Immulon 4HB microtiter plate wells were coated overnight at 4°C with SHP-2 (5 µg/mL) in TBS, pH 8 (coating buffer). The wells were then blocked with 5% BSA (EMD Millipore, 12659) in TBS, pH 8 for 1 h at room temperature. After washing the wells with TBS containing 0.05% Tween 20, the indicated concentrations of active PDGFRβ kinase domain (KD), sPDGFr or GST:LRP1-ICD were added to the wells in incubation buffer (TBS containing 0.5% BSA, 0.05% tween 20 and sodium orthovanadate), and incubated overnight at 4°C. Bound proteins were detected by addition of anti-GST HRP-conjugated antibody (Cell Signaling Technology, 5475) or anti-PDGFRβ Rabbit mAb (28E1, Cell Signaling Technology, 3169) followed by Goat anti-rabbit HRP-conjugated (Bio-rad, 170–6515). TMB microwell peroxidase substrate (KPL, 50-76-00) was added to the wells and the amount of bound ligand was measured spectrophotometrically at 620 nm. Data were analyzed by nonlinear regression analysis using Graphpad Prism 5 software employing the equation: *A = A_max_*/(1+ *K_d_*/[L])+B, where A = absorbance, Amax = maximum absorbance, K*_d_* = dissociation constant, L = ligand concentration and B = background absorbance.

### Malachite Green Phosphatase Assay

The catalytic activity of SHP-2 was assessed using a malachite green phosphatase assay kit (Echelon Biosciences). Recombinant SHP-2 (400 nM) was incubated with PDGFR KD (400 nM) or sPDGFRr (400 nM) or GST-LRP1-ICD (400 nM) in the presence of 1 mM ATP and 1 mM MgCl_2_. The substrate for SHP-2 was Src pY529 (TSTEPQ-pY-QPGENL), (Upstate biotech) and was used at a concentration of 250 µM. 25 uL of each sample were transferred to a 96 well Immulon 4HB microtiter plate, in duplicate. TBS buffer with 1 mM ATP and 1 mM MgCl_2_ with indicated proteins were assayed as a control. Malachite green solution (Echelon Biosciences) was added to each well and allowed to incubate for 15 minutes at room temperature. Liberated phosphate complexed with malachite green forms a colored complex. Absorbance was spectrophotometrically measured at 620 nm.

### Surface Plasmon Resonance

Binding of GST:LRP1-ICD to recombinant human SHP-2 (R&D Systems, 1894-SH-100) was measured using a BIA 3000 optical biosensor (BIAcore AB, Uppsala, Sweden). For these studies, a CM5 BIAcore sensor chip was activated, and GST:LRP1-ICD (either phosphorylated or unphosphorylated) was coupled as described [16]. An additional flow cell, similarly activated and blocked without immobilization of protein, served as a negative control. All binding reactions were performed in 10 mM HEPES, 0.15 M NaCl, 0.05% Tween 20, 2 mM sodium orthovanadate, pH 7.4 (HBS-P buffer) (BIAcore, AB). SHP-2 (1 µM) was injected and the binding was measured at 25°C at a flow rate of 20 µl/min for 1 min followed by 2 min of dissociation. The bulk shift due to changes in refractive index measured on blank surfaces was subtracted from the binding signal at each condition to correct for nonspecific signals.

### Endocytosis Assay

Murine aortic SMC (MOVAS) were grown in 6-well plates in DMEM supplemented with 10% fetal bovine serum. Cells were serum-starved for 24 h before the experiment. To block dynamin-mediated endocytosis, cells were treated with a small molecule inhibitor of dynamin, dynasore [28] (Santa Cruz Biotechnology). Cells were preincubated with 100 µM dynasore at 37°C for 30 min in serum-free conditions before the addition of 30 ng/ml human recombinant PDGF-BB (Cell Signaling Technology, #8912). Media was removed and cells were lysed and analyzed by immunoblot analysis with anti-SHP-2 antibody (Santa Cruz Biotechnology, sc-7384) and with phospho-SHP-2 y542 and y580 antibodies (Cell Signaling Technology, 3751 and 3703).

### Immunofluorescence Microscopy

WI-38 fibroblasts were serum starved overnight and then stimulated with PDGF-BB (30 nm/mL). Cells were serum-starved for 24 hours before adding PDGF-BB (30 ng/mL). The cells were incubated at 37°C for 10 min followed by washing in PBS and fixation in 3.7% formaldehyde, 5% sucrose in PBS for 20 minutes at room temperature. The fixed cells were permeabilized with 0.5% Triton X-100 for 5 minutes, blocked with 5% Donkey Serum for 1 hour, and then incubated with anti-phospho SHP-2 rabbit monoclonal (Santa Cruz) and mouse monoclonal 8G1 anti-LRP IgG (5 µg/mL) for overnight at 4°C. Slides were then washed and incubated with donkey Alexa-conjugated anti-rabbit and anti-mouse secondary antibodies (Invitrogen) for 1 h at room temperature. Stained coverslips were washed and mounted onto glass slides using FluorSave Reagent (Calbiochem) and viewed with laser scanning system Radiance 2100 (Zeiss/Bio-Rad) equipped with the argon/krypton (488/568 nm) and red diode (638 nm) lasers. The images were acquired and stored in RAW format using LaserSharp2000 software (Zeiss, Inc.). The images were exported into TIFF format and processed by Adobe Photoshop software (Adobe Systems) for publication. Adjustments of brightness and contrast were made for the entire image and were performed identically for both “single channel” and “merged” images.

### Chemotatic Assays

Migration experiments were performed with 24-well plates containing 5-µm pore size transwell filters (Costar). PEA-13 (LRP1^−/−^) and B41 clones (LRP1^+/+^) were seeded 2×10^4^ cells per filter onto the insert membrane and grown overnight. Cells were pretreated for 3 hours with 30 µM SHP-2 inhibitor, NSC-87877 (Millipore, 565851). Either buffer or PDGF-BB (30 ng/ml) was applied to the bottom chamber and incubation was carried out for 4 hours at 37°C. The topsides of filters were cleared of cells with a cotton swab. Cells were fixed with ice-cold methanol for 5 min and nuclei were stained with Vectashield mounting medium with Dapi (Vector Laboratories). Filters were mounted onto glass slides and fluorescent images of three random fields from two independent experimental membranes for each experimental condition were acquired on a Nikon E800 Eclipse microscope and quantified using software Volocity (Improvision).

### Statistical Analysis

Statistical analyses were performed using Graphpad Prism 5 software. Data are presented as means ± Std and were compared using a Student *t*-test or a one way Anova. Threshold for significance was set as p≤0.05. ELISA experiments were performed with triplicates, and each experiment was repeated twice.

## Results

### SHP-2 interacts with the Tyrosine-phosphorylated form of the LRP1 Intracellular Domain with High Affinity

**Figure 1:**
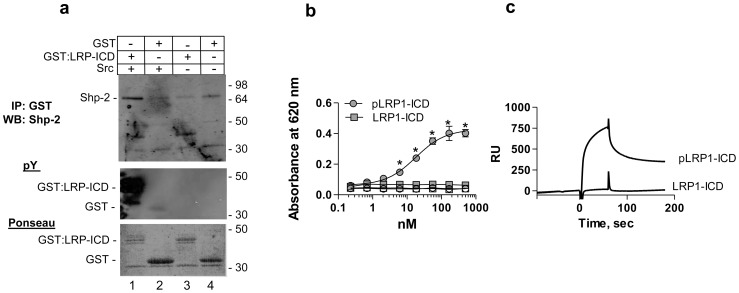
Phosphorylated LRP1-ICD interacts with SHP-2 with high affinity. (a) Cell lysates from NRK fibroblasts were incubated with phosphorylated GST:LRP1-ICD (*lane 1*), unphosphorylated GST:LRP1-ICD (*lane 3*), GST and src (*lane 2*) or GST alone (*lane 4*) all bound to Glutathione-Sepharose. Following incubation and washing, eluted proteins were separated by SDS-PAGE and analyzed by immunoblot analysis for SHP-2 (*upper panel*), for tyrosine phosphorylation (*middle panel*) and for total protein by Ponseau stain (*lower panel*). (b) Increasing concentrations of phosphorylated (circles) or unphosporylated (squares) GST:LRP1-ICD were incubated with microtiter wells coated with SHP-2 (closed symbols) or BSA (open symbols). Bound GST:LRP1-ICD was detected with anti-GST antibodies. Curve shows the best fit to a single class of sites using non-linear regression analysis. *, absorbance values for pLRP1-ICD are significantly different from those of LRP1-ICD (p<0.0001, Students t test) (c) SPR analysis confirms binding of SHP-2 (1 µM) to immobilized phosphorylated GST:LRP1-ICD but not to immobilized unphosphorylated GST:LRP1-ICD.

LRP1 contains two NPXY motifs within its ICD, one of which is preferentially phosphorylated on tyrosine (tyrosine 4507) by Src-family kinase members as well as the activated PDGFRβ [15], [16], [29], [30]. Tyrosine phosphorylation at tyrosine 4507 in the LRP1-ICD generates a docking site for a number of adaptor proteins, including Shc [18] and SHP-2 [19], [20]. To confirm the interaction between phosphorylated forms of the LRP1-ICD and SHP-2, NRK fibroblasts were grown to subconfluence and cell lysates were incubated with phosphorylated or unphosphorylated GST:LRP1-ICD bound to Glutathione-Sepharose. Following washing, immunoblot analysis of the bound proteins indicated that the phosphorylated form of GST:LRP1-ICD, but not the unphosphorylated form, associates with endogenous SHP-2 from fibroblasts (Fig. 1a).

To quantify the interaction of SHP-2 and the LRP1-ICD and to demonstrate a direct interaction between these two molecules, we performed an ELISA in which microtiter wells were coated with SHP-2 and titrated with increasing concentrations of phosphorylated or unphosphorylated forms of GST:LRP1-ICD. Following incubation and washing, bound GST:LRP1-ICD was detected with anti-GST antibodies. The results shown in Figure 1b, reveal that phosphorylated GST:LRP1-ICD bound to SHP-2 with a high affinity (K_D_ = 18+/−4 nM). In contrast, the unphosphorylated form of GST:LRP1-ICD failed to bind to SHP-2. As a control experiment, the binding of GST to SHP-2 immobilized on microtiter wells was also performed, and no binding was detected (data not shown). An additional control for this experiment measured the binding of GST:LRP1-ICD to BSA-coated wells, which confirmed no binding to BSA. Since ELISA based experiments measure high affinity interactions, to determine if unphosphorylated forms of the LRP1-ICD interacts weakly with SHP-2, we performed surface plasmon resonance experiments in which phosphorylated or unphosphorylated GST:LRP1-ICD was bound to SPR chips and SHP-2 was injected over these surfaces. The results of this study verified the interaction between SHP-2 and the phosphorylated form of GST:LRP1-ICD (Fig. 1c). In addition, the results reveal that the SHP2 fails to bind to unphosphorylated forms of LRP1-ICD (Fig. 1c).

### SHP-2 also Interacts with the Phosphorylated form of PDGFRβ Kinase Domain (KD) with High Affinity

**Figure 2:**
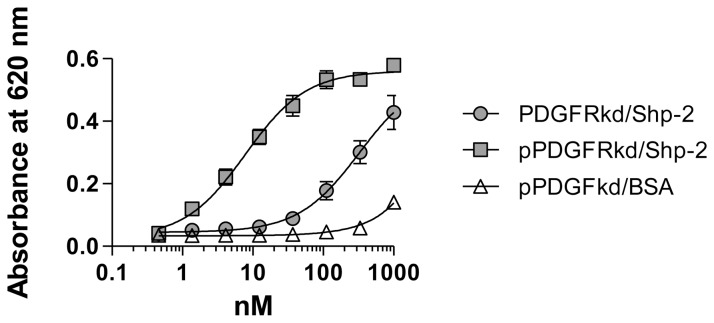
Quantitative analysis of interaction of the PDGFRβ kinase domain with SHP-2. Increasing concentrations of phosphorylated (closed squares) or unphosphorylated PDGFRβ KD (closed circles) were incubated with microtiter wells coated with SHP-2. Bound PDGFRβ KD was detected using an anti-PDGFRβ antibody. As a control, the binding of phosphorylated PDGFRβ KD to BSA (open triangles) was also measured. Curves show the best fit to a single class of sites using non-linear regression analysis. The binding of pPDGFRβ to SHP-2 is significantly different from the binding of PDGFRβ to SHP-2 (p = 0.0002, Students t test).

A specific interaction between SHP-2 and phosphorylated tyrosines 763 and 1009 in the cytoplasmic domain of the activated PDGFRβ has been demonstrated by co-immunoprecipitation analysis [22], [23]. To determine the affinity of this interaction, we performed an ELISA in which increasing concentrations of phosphorylated or unphosphorylated kinase domain of PDGFRβ (PDGFRβ KD) were incubated with microtiter wells coated with SHP-2 and bound PDGFRβ KD was detected with specific anti-PDGFRβ antibody. The results of this experiment are shown in Figure 2, and reveal a high affinity interaction between pPDGFRβ KD and SHP-2 (K_D_ = 7.8+/−1 nM). In contrast, a relatively weak interaction of non-phosphorylated forms of the PDGFRβ KD with immobilized SHP-2 was detected (K_D_ = 326+/−68 nM).

### PDGFRβ KD Competes with LRP1 for the Binding of SHP-2

**Figure 3:**
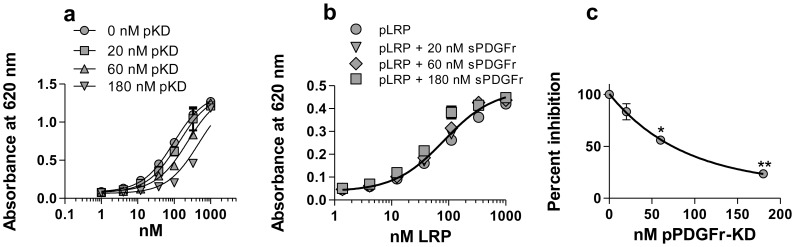
pPDGFRβ kinase domain and pLRP1-ICD compete for SHP-2 binding. (a,b) Increasing concentrations of GST:pLRP1-ICD were incubated with microtiter wells coated with SHP-2 in the presence of 0, 20, 60 and 180 nM pPDGFRβ KD (a) or sPDGFr (b). Bound GST:pLRP1-ICD was detected with anti-GST antibody. Curves in (a) show best fits to a single class of sites using non-linear regression analysis. The K_D_ values at each concentration are significantly different (p = 0.0007, Students t test). (c) Percent binding, normalized to 0 nM pPDGFRβ, of GST:pLRP1-ICD (100 nM) binding to SHP-2 in the presence of 20, 60 and 180 nM pPDGFRβ KD. (*, p = 0.0024; **p<0.0001, Students t test).

Since phosphorylated forms of LRP1 and PDGFRβ KD both bind to SHP-2 with high affinity, we conducted experiments to determine if these two receptors compete for the binding of SHP-2. To investigate this possibility, we incubated microtiter wells coated with SHP-2 with increasing concentrations of phosphorylated GST:LRP1-ICD in the presence of constant amounts of phosphorylated PDGFRβ KD. The results of this experiment (Figure 3a) demonstrate that in the presence of constant and increasing amounts of phospho-PDGFRβ KD, the apparent affinity of phosphorylated GST:LRP-ICD for SHP-2 decreases. As a control for these experiments, we performed the assay in the presence of soluble forms of the PDGFRβ ectodomain, and found no effect on the interaction of LRP1-ICD with Shp-2 (Fig. 3b). To examine more directly the impact of phosho-PDGFRβ KD on the ability of pLRP-ICD to bind SHP-2, the amount of binding of 100 nM pLRP-ICD to SHP-2 was plotted versus the concentration of p-PDGFRβ KD. These results (Fig. 3c) reveal that the pPDGFRβ KD competes with pLRP-ICD for binding to SHP-2.

### pPDGFRβ KD Directly Phosphorylates SHP-2

**Figure 4:**
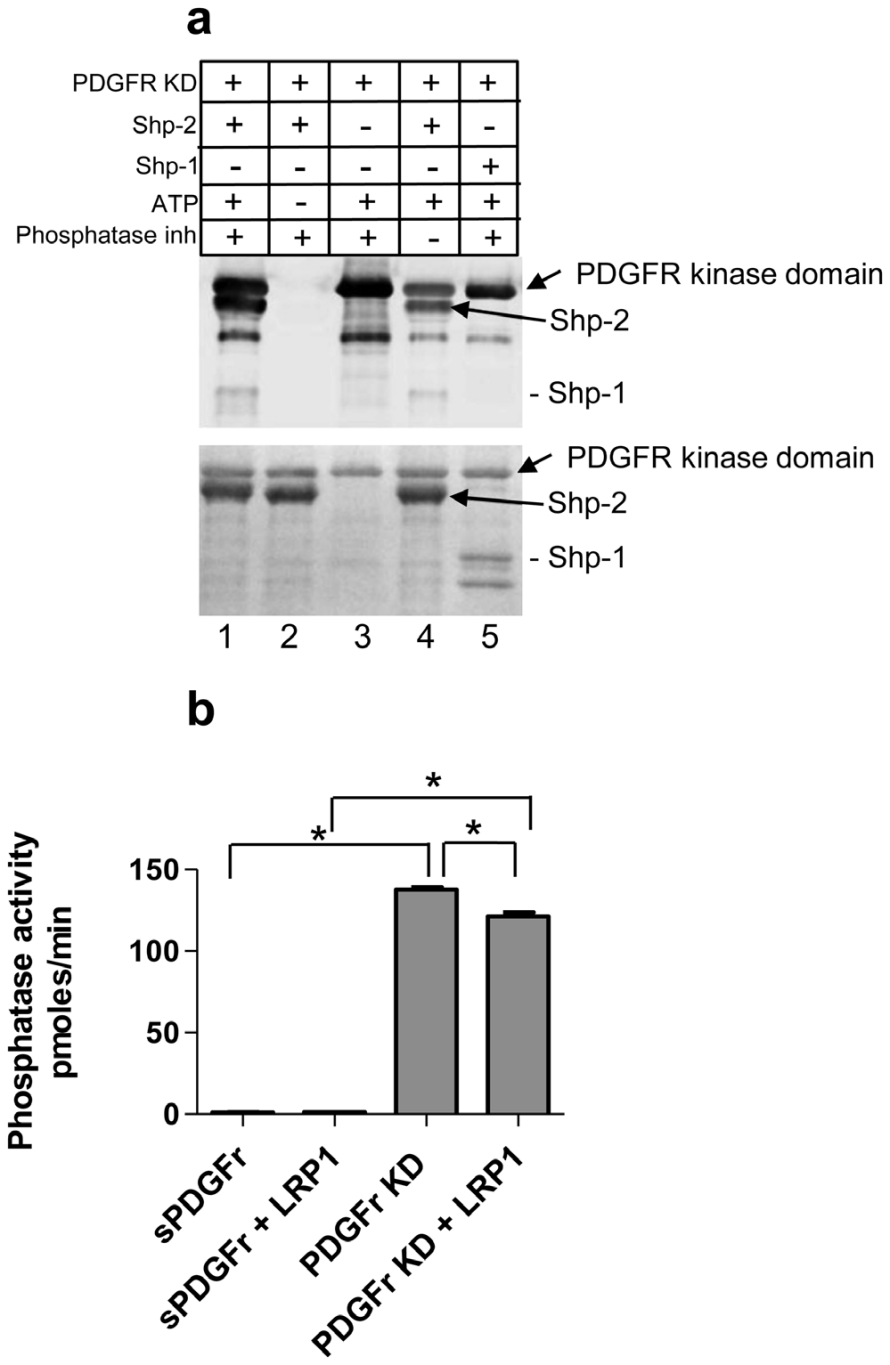
PDGFRβ kinase domain directly mediates the phosphorylation of SHP-2. (a) Purified PDGFRβ KD was incubated with recombinant human SHP-2 in the presence or absence of ATP and phosphatase inhibitors. Proteins were separated by SDS-PAGE and analyzed by immunoblot analysis for phosphotyrosine (*upper panel*). Total proteins are shown in the lower panel. As a control, SHP-1, a structurally similar phosphatase to SHP-2, was also included (lane 5). (b) The catalytic activity of SHP-2 was assessed using a malachite green phosphatase assay kit using Src pY529 (TSTEPQ-pY-QPGENL) as the substrate. Recombinant SHP-2 was incubated with pPDGFR KD or sPDGFRr, GST:LRP1-ICD, in the presence ATP and MgCl_2_. Liberated phosphate complexed with malachite green was measured at 620 nm (*p<0.0001 one way ANOVA5; *p<0.05 Tukey’s multiple comparison post hoc test).

SHP-2 has been reported to dephosphorylate the PDGFRβ at tyrosines 771, 751 and 740 [31], however it remains unclear whether activated PDGFRβ can directly mediate the phosphorylation of SHP-2. To examine this possibility, purified PDGFRβ KD was incubated with SHP-2 or the structurally related SHP-1, in the presence or absence of ATP and phosphatase inhibitors. Following incubation, proteins were separated by SDS-PAGE and the degree of protein phosphorylation was assessed by immunoblot analysis using an anti-phosphotyrosine antibody. Since autophosphorylation occurs between dimerized PDGF receptors, in the presence of ATP and phosphatase inhibitors the PDGFRβ KD is phosphorylated as predicted (Fig. 4, lane 3). In the presence of ATP and phosphatase inhibitors, both the PDGFRβ KD and SHP-2 are phosphorylated as a consequence of the PDGFRβ KD kinase activity (Fig. 4, lane 1). In the absence of ATP (Fig. 4, lane 2), the kinase activity of PDGFRβ KD is inactive and neither the PDGFRβ KD or SHP-2 are tyrosine phosphorylated. In the absence of phosphatase inhibitors, reduced phosphorylation of PDGFRβ KD and SHP-2 is noted (Fig. 4, lane 4). When PDGFR KD is incubated with SHP-1 in the presence of ATP and phosphatase inhibitors, the PDGFRβ KD was observed to be phosphorylated whereas SHP-1 was not (Fig. 4, lane 5). These results demonstrate that SHP-2, but not SHP-1, is directly phosphorylated by the activity of the PDGFRβ KD.

To determine the effect of SHP-2 phosphorylation by the PDGFRβ KD on the catalytic potential of SHP-2, we measured the phosphatase activity of SHP-2 after incubating with the PDGFRβ kinase domain in the presence of ATP. As a control for this experiment, we also used the soluble ectodomain of the PDGFRβ (sPDGFr). The results (Fig. 4b) demonstrate that SHP-2 phosphorylation by the PDGFRβ KD induces the catalytic activity of SHP-2. The presence of LRP1-ICD has little effect on SHP-2 phosphatase activity. However, the presence of LRP1-ICD (at equimolar concentration) reduces the activation of SHP-2 by the kinase domain of the PDGFRβ slightly (p<0.005), suggesting that LRP1 may reduce SHP-2 activation mediated by the PDGFRβ KD.

### PDGFRβ-mediated Tyrosine Phosphorylation of SHP-2 Occurs in Endosomes

**Figure 5:**
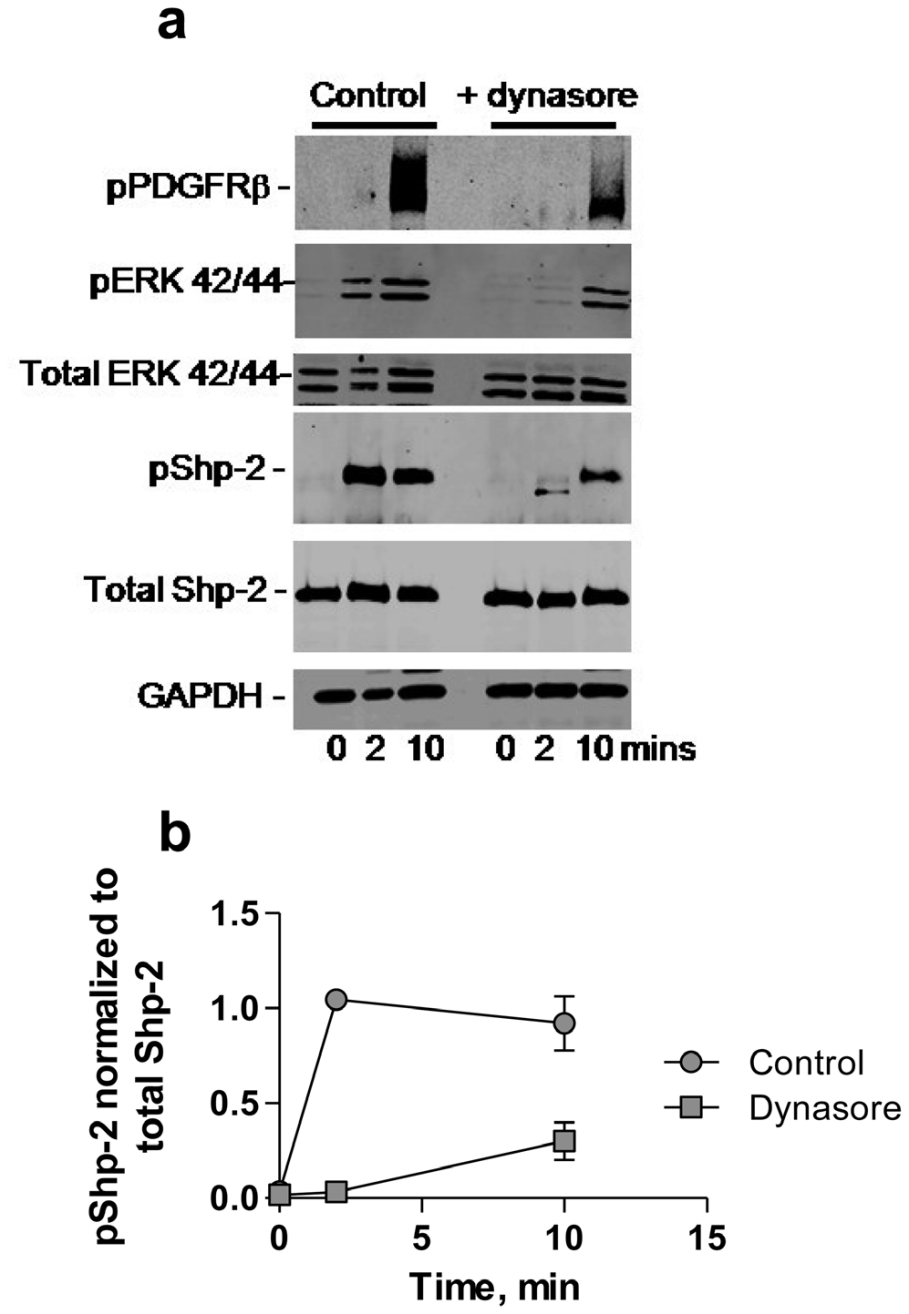
PDGF-mediated tyrosine phosphorylation of SHP-2 occurs within endosomes. (a) MOVAS cells were serum-starved and incubated with or without dynasore (100 µM) at 37°C for 30 min. Cells were then incubated with or without PDGF-B (30 ng/ml) for 2 and 10 min at 37°C. Cells were lysed and proteins separated by SDS-PAGE and analyzed by immunoblotting using the indicated antibodies. (b) Levels of phosphorylated SHP-2, normalized to total SHP-2 amounts in untreated (control) and dynasore treated cells are plotted.

Previous studies reveal that activation of the PDGFRβ results in tyrosine phosphorylation of LRP1 [16], [30] which can occur in caveolae [30] or within endosomal compartments following internalization of LRP1 and the PDGFRβ [17]. To explore the possibility that SHP-2 may also be activated by PDGF-mediated phosphorylation within endosomal compartments, we examined the effects of the dynamin-dependent endocytosis inhibitor, dynasore, on PDGFRβ-mediated SHP-2 phosphorylation. These results reveal that SHP-2 phosphorylation was delayed in cells that were treated with dynasore (Fig. 5), suggesting that SHP-2 phosphorylation is enhanced when PDGFRβ is trafficked to endosomes following its activation. Similar results are noted with ERK activation (Fig. 5) consistent with our previous studies [17].

### LRP1 co-immunoprecipitates and Co-localizes with Phosphorylated SHP-2 Following PGDF-BB Stimulation

**Figure 6:**
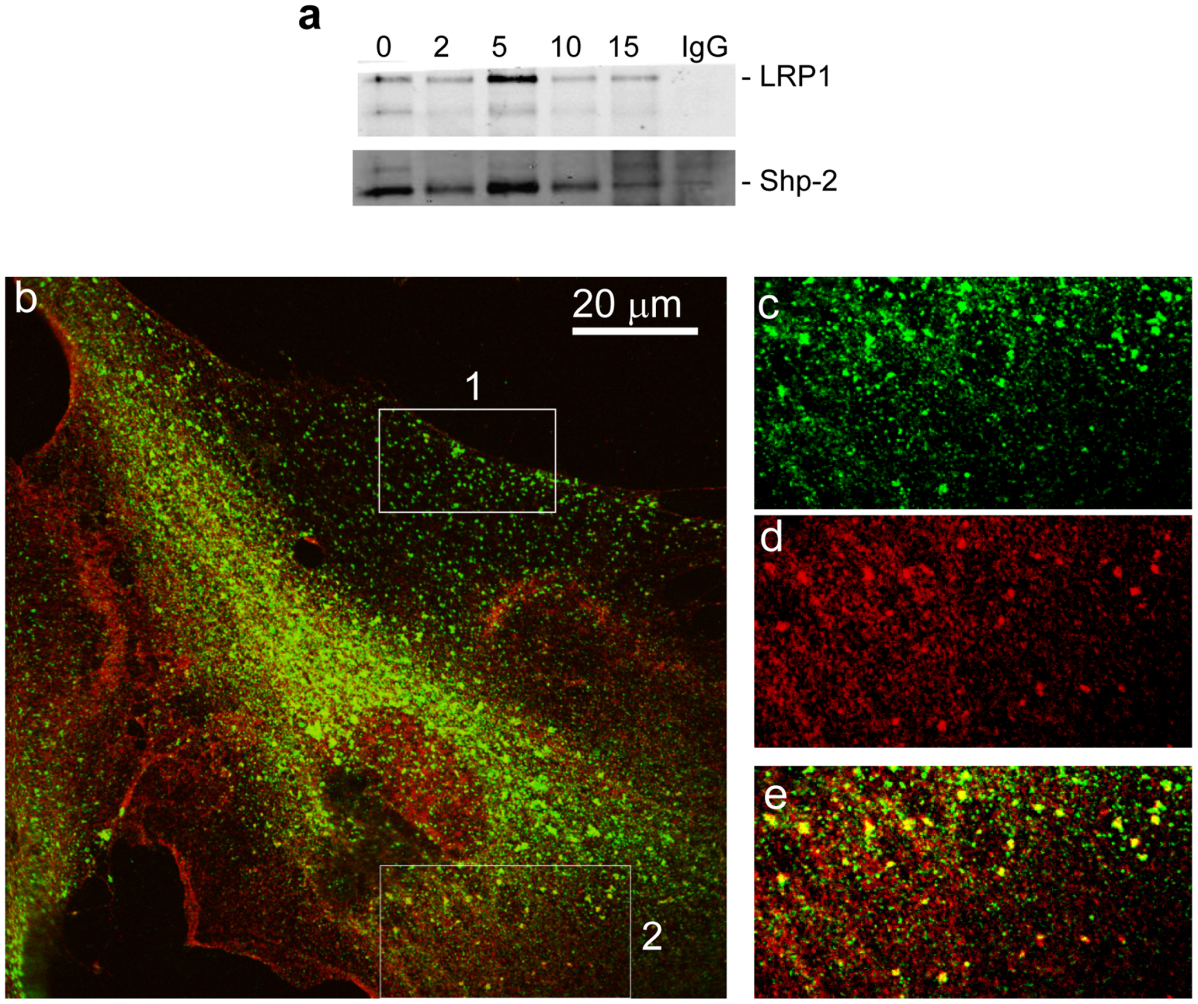
SHP-2 and LRP1 co-immunoprecipitate and co-localize in fibroblasts following PDGF stimulation. (a) WI38 fibroblasts were serum starved overnight and stimulated with PDGF (30 ng/mL) for 2, 5, 10 and 15 minutes at 37°C. Cells were treated with DSP crosslinker for 30 minutes on ice prior to lysis. Lysates were immunoprecipitated with a SHP-2 antibody (sc-280) and western blot analysis was performed using a monoclonal antibody to LRP1, 11H4 (top panel). Loading was controlled by using anti-SHP-2 IgG (bottom panel). As a control, non-immune IgG (IgG) was employed for immunoprecipitation (15 min stimulation with 30 ng/ml PDGF). (b) Immunflourescence studies reveal colocalization of LRP1 and phospho-SHP-2 in fibroblasts stimulated with PDGF-BB. WI38 cells were cultured on glass cover slips, fixed with formaldehyde, and processed for immunofluorescent microscopy as described in “Methods”. The confocal image was taken using 60X oil immersion objective. Box 1 is a representative section from the ‘trailing’ edge of this migrating cell and box 2 is a representative area of the ‘leading’ edge of the cell. (c–e) represent a 3 fold enlarged area from box 2, at the ‘leading’ edge of the cell; (c) *green* LRP1 staining, (d) *red* phospho-SHP-2 staining, (e) *yellow* colocalization of LRP1 and phospho-SHP-2.

**Figure 7:**
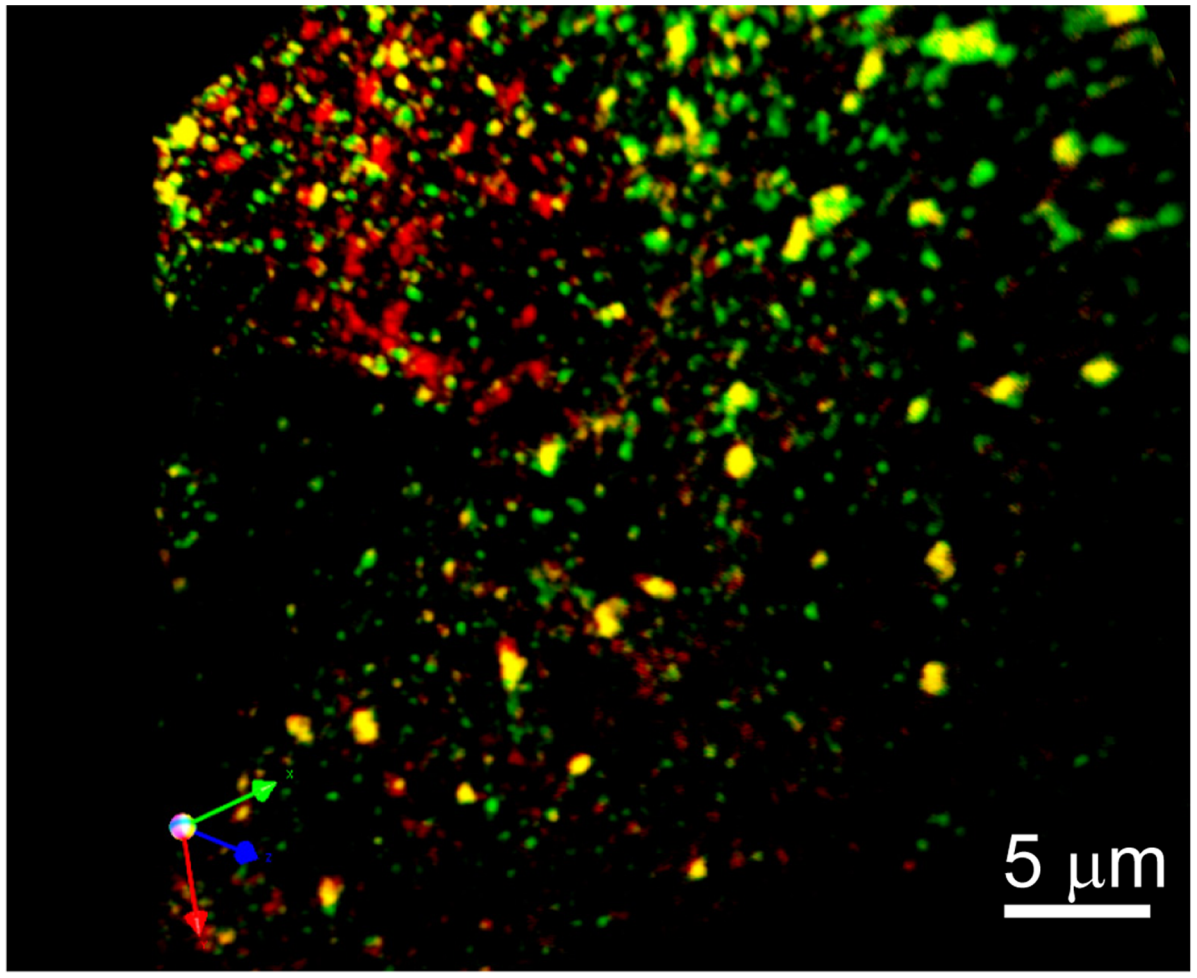
Co-localization of LRP1 and phospho-LRP1 in WI-38 fibroblasts. Three-dimensional reconstruction from a stack of optical sections of the leading edge of a cell depicted in Fig. 6 (box 2). Image is rendered to show sites of colocalization facing forward. Stacks of 5 images 0.2 µm apart were captured with a BioRad confocal (Zeiss) microscope using 100X oil immersion objective; 3D reconstruction was done using Volocity (Improvision) software. LRP1 (green) and phospho-SHP-2 (red).

To examine the possible formation of a complex between LRP1 and phospho-SHP-2, we utilized WI38 fibroblasts which express abundant levels of LRP1 and SHP-2, thus making them an optimal cell system in which to investigate associations between these two molecules. Following PDGF stimulation, cells were chilled and incubated with the cell-permeable crosslinking agent, DSP (dithiobis[succinimidylpropionate]) prior to analysis. The results (Fig. 6a) reveal that very little LRP1 co-immunoprecipitates with SHP-2 in resting cells. However, upon stimulation with PDGF, we note a time-dependent co-immunoprecipitation of LRP1 with SHP-2. Immunofluorescence studies performed prior to PDGFRβ activation revealed that there is virtually no co-localization between LRP1 and SHP-2 (data not shown). However, following PDGF stimulation, LRP1 co-localized with SHP-2 at the leading edge of migrating cells (Fig. 6b, box 2 and c-e), but not at the trailing edge of the migrating cell (Fig. 6b, box 1). Five sections of the image (0.2 µm apart) were used to reconstruct a three-dimensional image, and the results are shown in Fig. 7, where it is apparent that LRP1 and phospho-SHP-2 co-localize in large vesicles, most likely multi-vesicular bodies or lysosomes.

### LRP1 Modulates SHP-2-dependent PDGFBB-induced Migration

**Figure 8:**
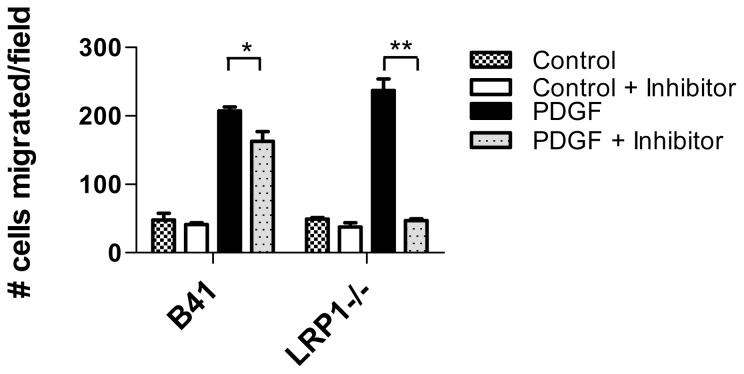
LRP1 modulates SHP-2-mediated migration in response to PDGF-B. LRP expressing (B41) and deficient (LRP^−/−^) fibroblasts were seeded onto 5 µm costar transwell filters at 2×10^4^ cells per well either in the presence or absence of the SHP-2 inhibitor, NSC-87877. After 3 h incubation at 37°C, either buffer (control) or PDGFB (30 ng/ml) was applied to the bottom chamber and incubation continued for 4 hours at 37°C. After incubation, the topsides of filters were cleared of cells with a cotton swab, and cells remaining on the bottom of the filters were fixed and the nuclei were stained with DAPI overnight. Filters were mounted onto glass slides and nuclei were quantified. (*p = 0.02, **p<0.0001, Students t-test).

SHP-2 is known to participate in PDGF-mediated chemotaxis, and to determine if LRP1 modulates the function of SHP-2 mediated chemotactic signaling, we examined the migration of LRP1-expressing (B41) and LRP1-deficient fibroblasts to PDGF-mediated chemotaxis. To examine the contribution of SHP-2 to this process, we employed the specific SHP-2 inhibitor, NSC-87877, which selectively inhibits the catalytic activity of SHP-2 [26]. The results of these experiments (Fig. 8) reveal that inhibition of SHP-2 phosphatase activity in LRP1 expressing cells has little overall impact on their chemotactic migration toward PDGF-BB. In contrast, inhibiting SHP-2 phosphatase activity in LRP1-deficient cells effectively blocks PDGF-BB chemotactic migration. These results suggest that in the absence of LRP1 expression, PDGF-mediated migration is dependent upon SHP-2. However, when LRP1 is expressed, PDGF-induced migration does not appear to depend upon SHP-2 activity.

## Discussion

Excessive vascular smooth muscle cell growth and migration are hallmark features of many vascular occlusive diseases including atherosclerosis and restenosis after percutaneous transluminal angioplasty (PTCA) [32]. These pathological responses to injury represent multicellular processes involving the local production of various growth factors, including PDGF. The PDGF signaling pathway plays a fundamental role in the development of vascular disease due to its ability to directly promote the transition of SMC from a quiescent, contractile state into a synthetic state of migration and proliferation [33]. Previous studies have shown that LRP1 is a physiological regulator of this pathway and functions to suppress PDGFRβ activation and signaling properties [13], [34]–[36]. The mechanisms by which this occurs are not at all clear. LRP1 can directly bind c-Cbl, a ubiquitin E3-ligase, that regulates turnover of receptor tyrosine kinases, such as the PDGFRβ. In fibroblasts from mice deficient in LRP1, ligand-induced PDGFRβ internalization and turnover is substantially increased [37]. However, these data do not explain the *in vivo* observations in smLRP1−/− mice where increased levels of active PDGFRβ and increased PDGF-signaling are observed [13].

Activation of the PDGFRβ upon binding of its ligand leads to a transient tyrosine phosphorylation of the LRP1-ICD [16], [30] which primarily occurs within endosomal compartments [17]. This event generates a docking site for adaptor molecules involved in signaling pathways, including Shc [18] and SHP-2 [19], [20]. SHP-2 is a non-receptor protein tyrosine phosphatase that functions as a positive signal transducer between receptor protein tyrosine kinases and the ERK pathway in mediating cellular responses. SHP-2 regulates the expression of the PDGFRβ, and thus levels of PDGFRβ are downregulated in mutant fibroblasts lacking SHP-2 [38]. SHP-2 is also directly involved in PDGF-mediated signaling events, and PDGF-stimulated DNA synthesis and ERK activation [38] as well as PI3K activation [38], [39] is severely suppressed in SHP-2 deficient cells. Activation of the PDGFRβ induces autophosphorylation of a number of tyrosine residues within the PDGFRβ cytoplasmic domain which act as docking sites for adaptor proteins. Two of these sites, located on tyrosines 763 and 1009, are responsible for binding SHP-2 [22], [23]. Interestingly, mutation of these two tyrosines to phenylalanine generates a receptor that fails to bind SHP-2 and demonstrates a significantly reduced chemotaxis response induced by PDGF-BB, revealing an important role for SHP-2 in chemotactic signaling [23], [40]. Together, these data reveal a critical function for SHP-2 in promoting the signaling responses of the PDGFRβ. Importantly, substantial evidence suggests an important role for SHP-2 in the vasculature. SHP-2 is abundant in vascular SMC [41], and its expression levels are elevated upon vascular injury [42], [43]. PDGF-mediated smooth muscle cell migration is inhibited by the SHP-2 inhibitor NSC-87877 and interestingly, oral administration of the SHP-2 inhibitor, NSC-87877 significantly suppressed neointima formation in a rat model of carotid artery injury [44].

Based on the important role of SHP-2 in facilitating PDGF-mediated signaling events, we hypothesized that the association of SHP-2 with phosphorylated forms of LRP1 may attenuate PDGF signaling events. To test this hypothesis, we first conducted experiment to measure the affinity of SHP-2 for phosphorylated forms of the LRP1-ICD and compared this value to its affinity for activated forms of the PDGFRβ. These studies confirmed high affinity interactions between SHP-2 and the phosphorylated forms of the LRP1-ICD that were comparable to those measured for its interaction with the PDGFRβ. Additional experiments demonstrated a competition between these two receptors for SHP-2. We conclude from these experiments that phosphorylated forms of LRP1 binds SHP-2 with an affinity that is comparable to that of the PDGFRβ, and is thus capable of sequestering SHP-2 and preventing its association with activated forms of the PDGFRβ. Interestingly, we confirmed that LRP1 co-immunoprecipitates and co-localizes with SHP-2 in fibroblasts following stimulation with PDGF. This co-localization occurs most prominently in large vesicles, likely multivesicular bodies. It is tempting to speculate that LRP1 may bind and sequester SHP-2 in multivescular bodies. Of interest in this regard are the recent studies of Taelman et al. [45] demonstrating that Wnt signaling involves sequestration of glycogen synthase kinase 3 inside of multivesicular endosomes.

In summary, findings in the present study demonstrate that LRP1 modulates SHP-2-mediated PDGFRβ signaling events. This occurs following tyrosine-phosphorylation of LRP1 that is mediated by activated PDGFRβ. Elucidating mechanisms which modulate PDGFRβ signaling will give a greater understanding of the process of vascular smooth muscle remodeling in the context of cardiovascular disease, and possibly other PDGFR signaling-driven processes such as cancer and pulmonary fibrosis.
